# Advances in the study of subclinical AKI biomarkers

**DOI:** 10.3389/fphys.2022.960059

**Published:** 2022-08-24

**Authors:** Chenchen Zou, Chentong Wang, Lin Lu

**Affiliations:** ^1^ Mudanjiang Medical College, Mudanjiang, Heilongjiang, China; ^2^ Mudanjiang Medical College, Mudanjiang, Heilongjiang, China; ^3^ Department of Integrative Medicine-Geriatrics, Hongqi Hospital, Mudanjiang Medical College, Mudanjiang, Heilongjiang, China

**Keywords:** acute kidney injury, subclinical acute kidney injury, biomarkers, early diagnosis, research progress

## Abstract

Acute kidney injury (AKI) is a prevalent and serious illness in all clinical departments, with a high morbidity and death rate, particularly in intensive care units, where prevention and treatment are crucial. As a result, active prevention, early detection, and timely intervention for acute kidney injury are critical. The current diagnostic criteria for acute kidney injury are an increase in serum creatinine concentration and/or a decrease in urine output, although creatinine and urine output merely reflect changes in kidney function, and AKI suggests injury or damage, but not necessarily dysfunction. The human kidney plays a crucial functional reserve role, and dysfunction is only visible when more than half of the renal mass is impaired. Tubular damage markers can be used to detect AKI before filtration function is lost, and new biomarkers have shown a new subset of AKI patients known as “subclinical AKI.” Furthermore, creatinine and urine volume are only marginally effective for detecting subclinical AKI. As a result, the search for new biomarkers not only identifies deterioration of renal function but also allows for the early detection of structural kidney damage. Several biomarkers have been identified and validated. This study discusses some of the most promising novel biomarkers of AKI, including CysC, NGAL, KIM-1, lL-18, L-FABP, IGFBP7, TIMP-2, Clusterin, and Penkid. We examine their performance in the diagnosis of subclinical AKI, limitations, and future clinical practice directions.

## Introduction

Acute Kidney Injury (AKI) is a serious hospital complication that compromises crucial illness. It is related with increase rates of short-term dialysis, morbidity, death rate, and prolonged hospitalization, and long-term negative output such as cardiovascular mortality and chronic kidney disease (Teo and Endre, 2017). Although renal tubular ischemia is the predominant mechanism of damage in AKI, it is frequently caused by multiple aspects including major surgery, sepsis, hypovolemia, poor cardiac output, urinary tract obstruction, rhabdomyolysis, and drug toxicity in clinical practice (Beker et al., 2018). Despite breakthroughs in AKI management, its morbidity and mortality remain high, owing primarily to unrecognizable kidney injury and delayed diagnosis. Relying on the patient’s underlying condition, the mortality rate of AKI patients ranges from 10% in hospitalized patients to 60% in intensive care unit (ICU) patients (Bhosale and Kulkarni, 2020). AKI also lengthens hospitalization, raises medical expenditures, and places a significant financial burden on patients (Rosner et al., 2018), and the prevention and treatment form is quite severe. Therefore, active prevention, early detection and timely intervention for acute kidney injury are vital.

## Limitations of creatinine (SCr) and urine output (UO)

The diagnosis of AKI remains highly complex, and multiple categorization criteria for diagnosing AKI have emerged, ranging from risk, injury, failure, renal function loss, end-stage renal disease (RIFLE), and the Acute Kidney Injury Network (AKIN) to the 2012 Kidney Disease Improving Global Outcomes (KDIGO) criteria. There are still limitations in using these criteria to diagnose AKI. Changes in SCr and UO represent only functional changes in the kidney. The increase in SCr concentration often comes after a large fall in GFR, and the renal tubular epithelium is the principal site of injury in most forms of AKI, SCr does not directly capture tubulointerstitial injury, and lacks accuracy in diagnosing structural renal damage (Moledina and Parikh, 2018). Furthermore, renal reserve, degree of tubular injury, intravascular volume status, patient muscle mass and nutrition, hemodynamic alterations, and fluid transfer all impact SCr levels (Bhosale and Kulkarni, 2020). Because creatinine accumulates in the body over time, acute alterations in GFR after renal injury do not lead in a rapid tise in SCr (Endre et al., 2011). After damage, it usually takes 24–36 h to achieve a steady-state (Waikar and Bonventre, 2009). The rise in SCr is slowed in patients with reduced GFR and associated fluid overload. Similarly, when GFR improves, SCr does not fall immediately (Chen, 2013). As a result, the lack of specificity, sensitivity, and timeliness limits serum creatinine levels. Urine output (UO) may be a time-sensitive marker of glomerular filtration rate (GFR), as it tends to decrease before serum creatinine concentrations increase (Rizvi and Kashani, 2017). However, in the absence of a catheter, UO is challenging to quantify and can be significantly affected by hypovolemic conditions and diuretics. Severe AKI can occur despite standard urine output. Fluid overflow is always seen in patients with critical AKI diagnosed by UO (Gameiro and Lopes, 2019). As a result, urine volume is insensitive, and non-oliguric AKI can develop with limited specificity (Srisawat and Kellum, 2020). Therefore, detection of AKI is greatly deferred in up to 43% of admitted patients, resulting in missed treatment windows.

## Subclinical AKI

**FIGURE 1:**
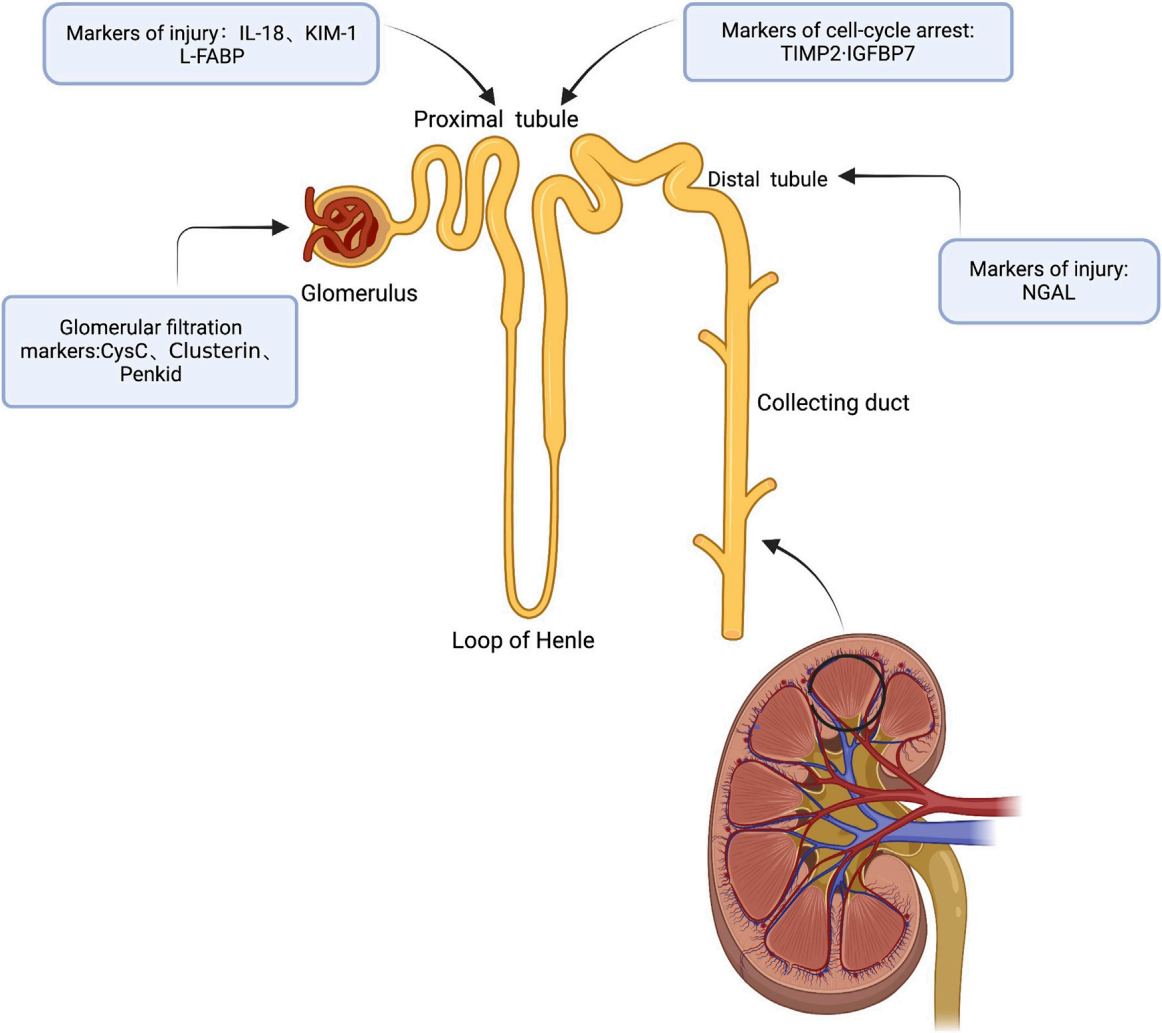
Subclinical AKI biomarkers are labeled according to anatomic location and/or production mechanism (Created with BioRender).

It is well known that the human kidney plays an important role as a functional reserve, and it is only when more than 50% of the kidney mass is compromised (i.e., GFR decreases by more than 50%) that dysfunction becomes apparent and SCr begins to rise. Because of this, observing changes in SCr may cause the clinical “window of opportunity” for treatment to be missed, especially in the acute stage. It is possible that the clinical “window of opportunity” for treatment will be missed if alterations in SCr are observed. This is especially during the acute tubular necrosis phase, as SCr does not differentiate between the many causes of AKI at this stage. AKI diagnosed only by elevated markers of tubular/glomerular injury is called subclinical AKI, and it is imperative to improve the recognition of subclinical AKI (Haase et al., 2012), when a clinical syndrome characterized by normal clinical SCr levels and normal or mildly decreased GFR occurs. Preclinical studies have repeatedly demonstrated that untreated acute tubular injury can lead to progression of AKI to chronic kidney injury (CKD), a finding that has been confirmed in patients with long-term follow-up AKI, and data from experimental and clinical studies have now established the role of tubular injury markers and demonstrated that these biomarkers can diagnose AKI early in the absence of other signals and clinical symptoms. This observation has also been validated in individuals with AKI who have been followed for a long time (Jones et al., 2012). Markers of renal tubular injury, particularly elevated levels of NGAL and KIM-1, which are associated with the transition from AKI to CKD, are specific indicators of high risk of progression to CKD. The study comprised of 1,635 patients from 3 centers (Nickolas et al., 2012), who were divided based on their blood creatinine level, KIM-1 level (>2.8 ng/ml) and NGAL level (>104 ng/ml). In the absence of acute renal function loss, a considerable proportion of patients (15%–20%) exhibited elevated markers of tubular injury, which increased the risk of death or requiring RRT by 2–3 times compared to patients without elevated levels of blood creatinine and markers of tubular injury (Haase et al., 2011). When patients have significant renal function loss, tubular injury marker levels can be tested to provide a prognostic assessment, with patients with two elevated tubular injury marker levels (NGAL, KIM-1) and elevated blood creatinine levels having the worst prognosis, with a 15.5%–17.5% mortality rate or need for RRT. Furthermore, some research suggests that tubular injury markers may fluctuate with time and length of injury, and that their levels represent the extent of injury. Based on these findings, ADQI proposes that the diagnosis of AKI be reformulated to include not only markers of renal function (e.g., changes in SCr and UO), but also markers of tubular injury such as NGAL, KIM-1, IL-18, and L-FABP (e.g., Figure 1), which, when combined, would not only better characterize the phenotype of AKI and enhance the diagnostic accuracy, but would also detect kidney damage prior to the rise of creatinine, which leads to the identification and treatment of subclinical AKI (Ostermann et al., 2020). In this study, we evaluate the most frequently investigated subclinical AKI biomarkers that are likely to be employed in clinical practice. We also summarize their performance in the diagnosis of subclinical AKI, as well as their current limits and future directions in clinical practice.

## Subclinical AKI biomarkers

### CysC

CysC is a cysteine protease inhibitor with a small molecular weight of 13 kDa, which allows it to be freely filtered in the glomerulus, disseminated on the cell surface, and about to completely reabsorbed and catabolized by proximal tubular cells (Yong et al., 2011). Given that the kidney is the sole organ capable of clearing CysC, it is a more accurate marker for determining glomerular filtration rate (GFR) than creatinine (Bhosale and Kulkarni, 2020). Compared to creatinine, CysC levels are not changed by age, sex, or muscle mass (Pozzoli et al., 2018). CysC performed substantially better than SCr and BUN in the mouse model of sepsis, with a three-fold increase in CysC at 3 h post sepsis compared to baseline (0 h) CysC. At this stage, the values of both SCr and BUN were twice as low as the baseline (Leelahavanichkul et al., 2014). It was discovered that an increase in CysC indicates tubular injury or damage and predicts AKI 24–48 h before an increase in SCr, suggesting that CysC can identify subclinical renal injury, in which renal injury occurs without a deterioration in renal function (Fang et al., 2018). In crucially ill patients, it is crucial to analyze patients with subclinical AKI who have renal damage but typical SCr levels. It is attainable to avoid the enlargement and growth of acute renal insufficiency if a timely diagnosis is made and preventative measures are adopted. Among the early diagnostic markers, CysC is a promising indicator. A recent study revealed that ICU patients who presented with elevated CysC levels at admission were more likely to develop AKI during their hospitalization. ROC curve analysis revealed that the AUC of Cys-C for predicting AKI was 0.67. With a sensitivity of 63% and a specificity of 66%, 0.94 mg/L was established to be the Cys-C threshold level (Gaygısız et al., 2016). Fang et al. discovered that CysC detects subclinical AKI in critically ill infants and children with an AUC of 0.72 and a sensitivity of 61.1% and specificity of 76.0% for peak uCysC, strengthening the support link between high CysC and AKI. CysC levels are a delicate indicator of subclinical AKI, and CysC-positive subclinical AKI is related to bad clinical results in crucially ill infants and children. Additional randomized and controlled clinical tests are required to determine if treating subclinical AKI improves clinical results in this populace (Fang et al., 2018). In a study of patients over 60 years old, Dalboni et al. found a contrary conclusion that Cys-C did not predict AKI, but greater Cys-C levels were an independent risk factor for death (Dalboni et al., 2013). Several investigations have found that Cys-C levels can alter as a result of conditions other than renal filtration (e.g., use of glucocorticoids, thyroid hormones, and systemic inflammation), however, acute inflammation, especially that induced by sepsis, has no effect on it (Manetti et al., 2005; Stevens et al., 2009). These studies have significant limitations, and the idea of CysC-positive subclinical AKI has yet to be verified in other case mixes, particularly in patients with high disease severity scores. More multicenter prospective studies are also needed to corroborate CysC’s age-related reference value (Fang et al., 2018).

### KIM-1

KIM-1 is a 38.7 kDa type I transmembrane glycoprotein (Oh, 2020) with immunoglobulin and mucin structural domains (Ichimura et al., 1998). In recent years, KIM-1 has been utilized as a sensitive indicator for the early detection of renal tubular injury with distinct advantages: in normal renal tissues, it is hardly expressed, however, its expression is significantly increased in mouse models of ischemia-reperfusion injury and drug-induced AKI, and KIM-1 is upregulation primarily in rodent and human S3 segments, where it is inserted into the apical membrane of the proximal tubule and remains in epithelial cells present until recovery (Vaidya et al., 2008). Furthermore, it is highly specific, particularly for ischemic or nephrotoxic AKI, rarely expressed in other organs, unaffected by pre-renal azotemia, urinary tract infections and chronic kidney disease (Assadi and Sharbaf, 2019). Moreover, KIM-1 has a protein hydrolysis area. It is easily detectable in urine, where it increases within the first hour following tubular toxicity or ischemic injury, far before serum creatinine (Lei et al., 2018; Pietrukaniec et al., 2020). Urinary KIM-1 can be used to differentiate patients with acute tubular necrosis from those with non-acute tubular necrosis, and measuring uKIM-1 levels in patients without AKI can serve as a marker of early kidney injury and predict adverse clinical outcomes, such as dialysis requirements and mortality (Kashani et al., 2017). KIM-1 can detect patients with subclinical AKI, who are at a higher risk of unfavorable consequences (Pietrukaniec et al., 2020). KIM-1 in urine has the potential to be a helpful biomarker for subclinical AKI associated with obstruction, according to Olvera-Posada et al. The significance lies not only in the ability to identify an obstruction marker in urine but also in the ability to quantify the grade of renal impairment to modify the treatment paradigm (Olvera-Posada et al., 2017). In a comparison of three promising urinary AKI biomarkers, KIM-1, NGAL, and IL-18, urinary KIM-1 had the maximal diagnostic performance in the initial diagnosis of AKI in children with hypovolemia, cardiogenic or aseptic shock before alterations in SCr became apparent (Assadi and Sharbaf, 2019). Urinary KIM-1 levels correspond with the amount of renal tissue injury and can be used to predict adverse renal outcomes in patients with acute tubular injury (ATI). Furthermore, correlating uKIM-1 and sKIM-1 can enhance the sensitivity and specificity for the detection of serious ATI, allowing clinicians to treat earlier, particularly in patients who are highly suspected of having ATI but are not appropriate for kidney biopsy (Cai et al., 2019). KIM-1 is approved in North America for preclinical monitoring of nephrotoxicity in drug development studies (Moledina et al., 2017). In a recent study, patients with mild and moderate COVID-19 who did not meet the requirement for AKI presented with findings of proximal tubular injury, particular non-albuminuria, and elevated uKIM-1 levels, indicating the emergence of subclinical AKI, in this study, the AUC was 0.830, with a sensitivity of 77% and a specificity of 76% (Yasar et al., 2022). However, some studies have found that comorbidities such as diabetes, hypertension, and atherosclerotic cerebral ischemia can have a significant impact on uKIM-1 concentrations. KIM-1 value is also susceptible to inflammatory diseases. All of these factors would reduce the specificity of AKI prediction.

### NGAL

NGAL is a 25 kDa protein that belongs to the lipocalin superfamily. Human NGAL was initially recognized from the supernatant of activated neutrophils (Kjeldsen et al., 1993) that are usually the primary cellular source of circulating NGAL. Physiologically, NGAL binds to iron-siderophore complexes, limiting bacterial iron uptake, and by sequestering iron-siderophore complexes, NGAL intercede the mitogenic impact of epidermal growth factor receptor (EGFR) signaling (Flo et al., 2004; Barasch et al., 2016). EGFR activation is related with stimulation of hypoxia-inducible factor-1α (HIF-1α) and NGAL expression, resulting in increased cellular proliferation, cytogenesis, and renal injury (Viau et al., 2010). NGAL is conveyed at reduce levels in different cell types, including prostate, uterus, salivary gland, trachea, lung, stomach, kidney, and colon (Friedl et al., 1999). It is the most extremely researched AKI biomarker, which is reabsorbed by the proximal tubules and released by the damaged distal tubules in the setting of acute tubular injury and can be identified within hours of tubular injury, even in lack of functional AKI (Lupu et al., 2021). NGAL was discovered to be one of the fastest growing genes in the kidney early after tubular injury, particularly in the distal tubular segment in transcriptome analysis studies in rodent models, and there is evidence that it may be the earliest known marker of renal injury. In mice, NGAL levels in the urine are significantly higher within 2 h after renal ischemia-reperfusion injury (Zhang and Parikh, 2019), while in humans, elevated NGAL levels can increase within 3 h after tubular injury and peak around 6–12 h, depending on the severity of the injury (Parikh et al., 2011). According to new research, elevated NGAL in urine can detect AKI as early as 2 h (Karademir et al., 2016). Haase et al. discovered that high NGAL levels were related with poor results even in the lack of diagnostic SCr elevations in a pooled data analysis of 10 studies of patients hospitalized to ICU (Casas-Aparicio et al., 2022). Urine NGAL was shown to detect NSAID-mediated renal tubular injury in the initial phase of renal injury in a cohort study of children with congenital heart disease who underwent cardiopulmonary bypass (CPB), and urine NGAL had good diagnostic accuracy in identifying children receiving NSAIDs, with an AUC of 0.95–0.96 at 24–48 h after administration of NSAIDs. Continuous NGAL monitoring in children using NSAIDs would allow doctors to identify subclinical AKI and its progression as depicted by elevated NGAL levels and would offer a therapeutic time window to prevent AKI and functional impairment (Nehus et al., 2017). NGAL is by far the most extensively characterized and researched biomarker of AKI in patients undergoing cardiac surgery, and it is also the focus of current research. NGAL demonstrated good diagnostic ability in predicting pediatric (AUC:0.96) (Dent et al., 2007) and adult cardiac surgery (AUC:0.72) (Ho et al., 2015). Patients are frequently administered diuretics, angiotensin-converting enzyme inhibitors, and antibiotics as needed following cardiac surgery (CSA), which may exacerbate the identified acute tubular injury. CSA-NGAL score can help avoid this from happening. Preoperative risk assessment can be aided by baseline measurements, and an elevated NGAL value may even suggest delaying surgery until renal function can be maximized and additional injury avoided. The CSA-NGAL score, in addition to the functional score of AKI, can be used in prospective studies of patients having cardiac operations to identify subclinical AKI early and to take relevant interventions to prevent further injury or the development of functional AKI (de Geus et al., 2016). The performance of NGAL has also been studied in other contexts, including critically ill patients (AUC: 0.80) (de Geus et al., 2011), emergency department patients (AUC: 0.95) (Nickolas et al., 2008), and renal transplant patients (AUC: 0.82) (Pajek et al., 2014). In a cohort analysis of COVID-19 patients, urinary NGAL >150 ng/ml anticipated the duration, diagnosis, and severity of AKI and acute tubular damage, and admission, dialysis, shock, and death in patients with acute COVID-19 (Xu et al., 2021). NGAL was also discovered to be an independent risk factor for COVID-19 patients (He et al., 2021). In another cohort study, those with NGAL ≥45 ng/ml had a substantially shorter time to AKI than those with <45 ng/ml, but NGAL was not a risk factor for AKI during admission, and NGAL performed considerably better on day 7 than throughout hospitalization, implying that NGAL has a limited time window for predicting AKI and that higher NGAL thresholds appear to help predict the progression of AKI rather than the onset of AKI (Casas-Aparicio et al., 2022). Because NGAL threshold value is still controversial, including NGAL in clinical prediction models to improve disease identification requires additional research.

### IL-18

IL-18 is an interleukin-1 family pro-inflammatory cytokine generated by monocytes/macrophages and other antigen-presenting cells. IL-18 functions as an inactive precursor and is synthesized by a variety of tissues, such as monocytes, macrophages, proximal tubular epithelial cells, and intercalated duct cells of collecting ducts (Gauer et al., 2007). It is found intracellularly, and it is converted to the active form by caspase-1 (Schrezenmeier et al., 2017). IL-18 is an inflammatory mediator that is increased in numerous endogenous inflammatory processes, and its concentration is elevated in serum during sepsis, joint inflammation, inflammatory bowel disease syndrome, liver inflammation, and lupus (Beker et al., 2018). It is created in response to ischemia in different organs, including the kidney, heart, and brain. IL-18 levels in the kidney are enhanced in the presence of ischemia in animal experiments, and it has been demonstrated to be a mediator of acute tubular injury, inducing tubular necrosis through mediating ischemia-reperfusion injury and infiltration of neutrophils and monocytes into the renal parenchyma (Melnikov et al., 2001). IL-18 levels begin to rise roughly 6 h after kidney damage and peak between 12–18 h. Surprisingly, mice lacking IL-18 are protected from AKI caused by ischemia-reperfusion injury. Urinary IL-18 levels were considerably greater in patients with acute tubular necrosis in comparison to healthy subjects in subsequent trials (Parikh et al., 2004). According to a current systematic analysis of 11 studies in 2,796 patients, IL-18 is considered a promising biomarker with some diagnostic accuracy in the initial diagnosis of AKI, the AUC was 0.77 (95%CI 0.71–0.83) (Lin et al., 2015). In a study of AKI after CPB, urinary IL-18 levels were found to be more diagnostic than SCr and urinary NGAL in early diagnosis of AKI in clinical practice. At 2 h after CPB, the AUC was 83.25%, and when the threshold was set at 100 μg/L, the sensitivity and specificity were 90.91 and 93.83%, respectively (Wang et al., 2017). Isocyclophosphamide is a common nephrotoxic chemotherapeutic agent, and urinary IL-18 has to promise as a diagnostic test for early AKI in children treated with isocyclophosphamide and may play a possible role in drug toxicity monitoring (Sterling et al., 2017). As a result, it is an appealing target for biomarker-directed therapy for AKI, and more research on anti-IL-18 therapy is pending. Only a few clinical trials appear to have investigated the use of IL-18 as a biomarker for AKI (Lin et al., 2015). These studies have revealed acceptable outcomes in pediatric AKI patients following cardiac surgery (AUC:0.82) (Krawczeski et al., 2011; Zheng et al., 2013). Other studies, however, have found that IL-18 is not a strong predictor of AKI in ICU(AUC:0.59) or emergency department populations (AUC:0.64) (Nickolas et al., 2012; Nisula et al., 2015). According to a current systematic analysis, these inconsistencies may be resulted from the absence of clear consensus on the appropriate cutoff level of IL-18 for AKI prediction (Lin et al., 2015).

### L-FABP

L-FABP is a 14 kDa protein that belongs to the fatty acid-binding protein (FABP) family (Tan et al., 2002). It was discovered in the liver, where serum L-FABP levels are elevated in liver dysfunction (Pelsers et al., 2005), and it is also expressed in the kidney, intestine, pancreas, lung, and stomach. L-FABP has a high affinity and binding capacity for long-chain fatty acid oxidation products, suggesting that it could be a potent endogenous antioxidant (Ek-Von Mentzer et al., 2001). The transcriptional regulatory region upstream of the human L-FABP gene comprise transcription aspect confining areas related to ischemia and lipid metabolism, including hypoxia-inducible factor-1 (HIF-1), hepatocyte nuclear factor (HNF-1, HNF-4) and peroxisomal response element (PPRE), and gene expression is activated in response to ischemia and oxidative stress (Divine et al., 2003; Schachtrup et al., 2004). Furthermore, L-FABP binds to fatty acids and transports them to mitochondria or peroxisomes, participates in intracellular fatty acid homeostasis, and is one of the fundamental regulators of free fatty acid (FFA) stability *in vivo* (Martin et al., 2005). Excessive aggregation of FFA in the proximal tubules of the kidney, as well as its oxidation and peroxidation products, can cause enhanced tubular damage and high expression of L-FABP in renal tubular epithelial cells under stressful conditions (McMahon and Murray, 2010). Moreover, elevated levels of L-FABP in urine and plasma have been linked to the degree of renal injury (Kokkoris et al., 2013). In a cisplatin-induced kidney damage model in mice, urinary L-FABP levels rose before creatinine level elevation over 72 h. Noiriet revealed that urinary L-FABP increased 1 h after ischemia and that urinary L-FABP was superior to BUN and urinary NAG in initial and correct diagnosis of acute tubular necrosis in distinct AKI animal models (Noiri et al., 2009). Urinary L-FABP levels were substantially changed after cardiac catheterization in a study on contrast-induced AKI after cardiac catheterization in cardiac patients, but urinary NGAL, IL-18, and KIM-1 levels were not substantially changed, so this research determined that urinary L-FABP could be one of the helpful markers for detecting subclinical AKI due to contrast after cardiac catheterization (Hwang et al., 2014). Urinary L-FABP concentration was discovered by Portilla et al. to be utilized as an early and sensitive predictor of AKI complicating pediatric postoperative cardiac surgery, a 24-fold increase in urinary L-FABP at 4 h postoperatively, an AUC of 0.810, sensitivity of 0.714 and specificity of 0.684 (Portilla et al., 2008). Tang et al. (2017) also showed that urinary L-FABP was considerably elevated early in AKI (2 h postoperatively); utilizing SCr to diagnose AKI requires waiting until 48–72 h postoperatively, whereas applying 2-h postoperative urinary L-FABP for prediction. The utilization of Urine L-FABP at 2 h postoperatively can greatly advance the diagnosis of AKI. In predicting AKI, the AUCs of 2 and 6 h postoperative urinary L-FABP were 0.921 and 0.896, respectively. Meanwhile, urine samples were simple to collect and analyze, implying that elevated urinary L-FABP levels in the early postoperative period could better predict the occurrence of AKI. The combined application of urinary L-FABP and NGAL at 2 and 6 h postoperative cardiac surgery predicted the occurrence of AKI with AUC of 0.942 and 0.929, respectively, indicating that the combined test can more accurately predict the AKI’s occurrence and has a greater clinical value in the early diagnosis of AKI after cardiac surgery in children. Urine L-FABP has also demonstrated good performance in predicting AKI after cardiac surgery in adults, AUC of 0.720, and the application of urine L-FABP/creatinine ratio can improve urine L-FABP discrimination even more. Related research has also demonstrated that using extracorporeal circulation (CPB) time can help recognize people at high risk of AKI in cardiovascular surgery. CPB time is firstly used to assess the risk of postoperative AKI, afterwards, urinary L-FABP or urinary L-FABP/creatinine ratio was used to predict and diagnose AKI early, with urinary L-FABP at 16–18 h demonstrating improved discrimination with an AUC of 0.742, enabling more cost-effective and reliable risk identification than utilizing urinary L-FABP tests (Lee et al., 2021). In addition, urinary L-FABP levels at admission are a powerful predictor of long-term negative results in medical cardiac intensive care unit (CICU) patients. Urinary L-FABP may substantially improve long-term risk stratification in patients hospitalized to the medical CICU when combined with creatinine-defined AKI (Naruse et al., 2020). Moreover, baseline urinary L-FABP levels are a reliable biomarker for predicting AKI in patients with acute decompensated heart failure (ADHF), AUC was 0.930, sensitivity was 94.2%, specificity was 87.0% (Hishikari et al., 2017). However, certain existing renal diseases, including non-diabetic chronic kidney disease, early diabetic nephropathy, polycystic kidney disease, and idiopathic focal glomerulosclerosis, may affect the diagnostic performance of urinary L-FABP. Since L-FABP is conveyed in the liver, urinary L-FABP may lose its specificity for kidney disease when liver disease is also present. To summarize, L-FABP seems to be the best biomarker for the initial prediction of AKI (Cho et al., 2013), but its potential value must be verified in large-scale research and a broader clinical setting.

### TIMP-2·IGFBP7

**FIGURE 2:**
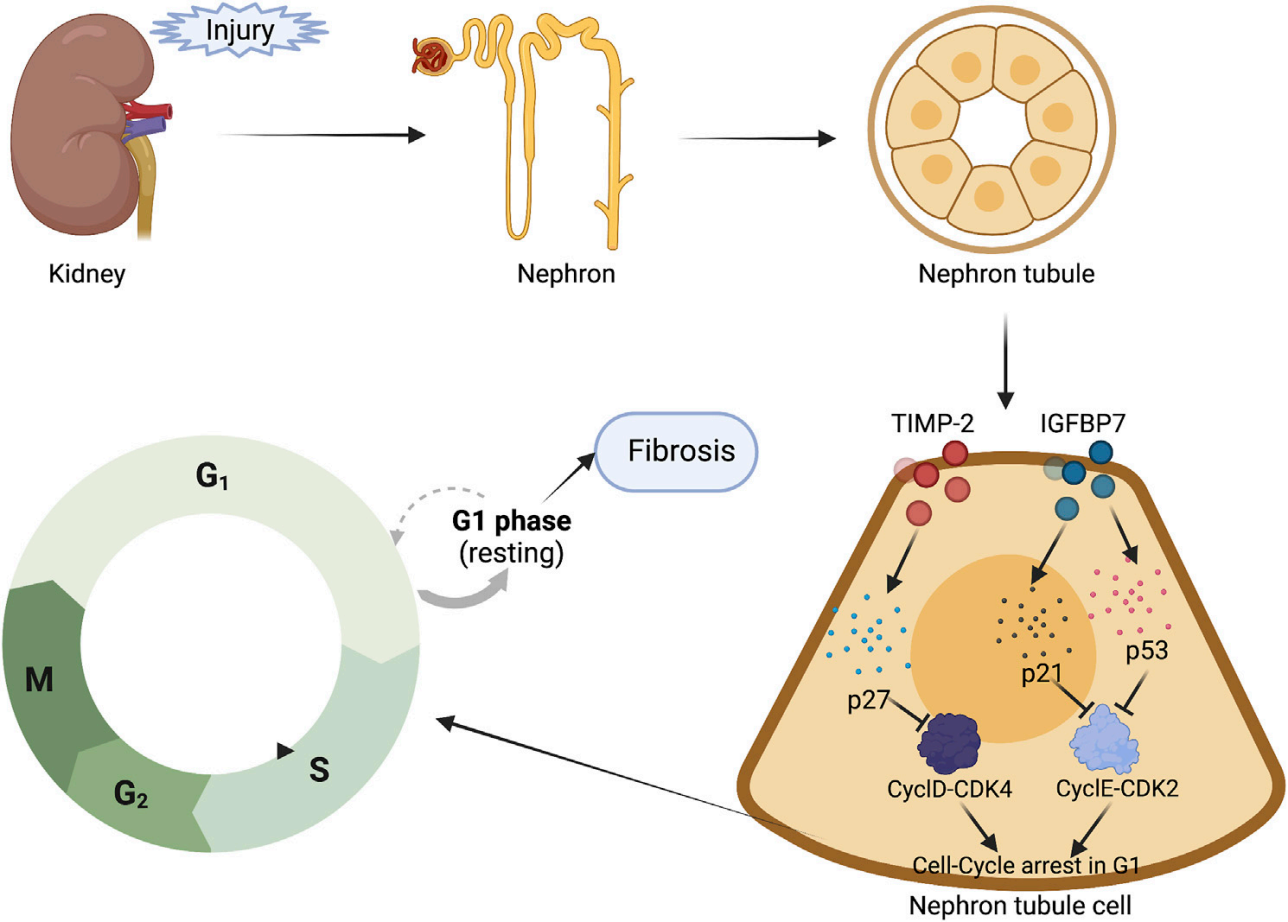
G1 Cell cycle arrest markers: TIMP-2 and IGFBP7(Created with BioRender).

TIMP-2 is a 21 kDa protein with anti-apoptotic and pro-proliferative properties, while IGFBP7 is a 29 kDa protein which acts as an IGF-1 receptor antagonist that causes tumor suppression and regulates cellular aging by inhibiting kinase signaling. Renal tubular cells express and secrete them (Bhosale and Kulkarni, 2020). TIMP-2 and IGFBP7 are both inducers of G1 cell cycle arrest that are produced during the early phases of cellular stress or injury (Cavalcante et al., 2022). Some tumor suppressor proteins such as p27, p53, and p21 are activated and upregulated differently after renal injury (e.g., oxidative stress, toxins, ischemia, sepsis, inflammation, etc.) (Ortega and Heung, 2018), with IGFBP7 directly increasing the expression of p53 and p21, while TIMP-2 enhances the expression of p27. These p-proteins prevent the cell cycle-dependent protease complexes (CyclD-CDK4 and CyclE-CDK2) from supporting the cell cycle progression, leading in transitory G1 cell arrest. This process inhibits cell division and apoptosis, allowing the cell to restore DNA damage and function (as shown in Figure 2) (Barnum and O’Connell, 2014). A prolonged arrest of tubular cells in G1 phase would lead in a senescent cell phenotype and fibrosis (Yang et al., 2010). TIMP-2 and IGFBP7 can signal in an autocrine and paracrine manner to send alarms from the location of injury to other sites (Ortega and Heung, 2018). The consolidation of these two biomarkers has been explored as predictors of AKI and is beneficial in identifying early structural kidney impairment, also known as subclinical kidney injury, which can indicate an increased risk of adverse outcomes. In a laboratory investigation in rats, the combination of TIMP2 and IGFBP7 was more accurate for detecting AKI than either marker alone. TIMP2-IGFBP7 was significantly raised in >10% of patients in the low-risk CI-AKI population, indicating structural kidney injury, while serum creatinine remained silent, thus revealing subclinical CI-AKI (SCI-AKI), according to a study on contrast-induced AKI (CI-AKI). The use of appropriate biomarkers early in the diagnostic process has the potential to assist clinicians in identifying patients with renal stress and injury for early observation or preventive intervention to restrict the development of AKI (Breglia et al., 2020). A significant proportion of COVID-19 severe illness patients are admitted with subclinical symptoms of renal dysfunction that has not yet constituted AKI. This study discovered that urinary TIMP-2-IGFBP7 ≥0.2 (ng/ml) 2/1000 was a risk factor for AKI, and individuals with higher TIMP-2-IGFBP7 had a significantly shorter time to AKI. TIMP-2-IGFBP7, when combined with clinical information, was an excellent predictor of subclinical AKI in crucially ill COVID-19 patients (AUC: 0.682) (Casas-Aparicio et al. 2022). Meersch et al. measured TIMP2-IGFBP7 concentrations in serial urine samples from 50 patients undergoing cardiac surgery; changes in creatinine and urine volume did not occur until 1–3 days postoperatively, whereas TIMP2-IGFBP7 concentrations began to rise as early as 4 h postoperatively in patients who had AKI, and the 4-h postoperative cutoff value demonstrated positive sensitivity and specificity, with an area under the ROC curve of 0.81 (CI: 0.68–0.93). Furthermore, a decrease in urine [TIMP-2] and [IGFBP-7] at discharge was a strong predictor of renal recovery (Meersch et al., 2014). Oezkur et al. measured TIMP-2-IGFBP-7 in urine samples at baseline, at ICU admission and 24 h following surgery with a cutoff of 0.3. The main endpoint was the occurrence of AKI within 48 h postoperatively. [TIMP-2] x [GFBP-7] values >0.3 were highly linked with the development of AKI (OR 11.8, *p* < 0.001) upon ICU admission, with a sensitivity of 0.60 and specificity of 0.88, whereas measurements before surgery (baseline) were not associated with the risk of AKI (Oezkur et al., 2017). Meersch et al. (2017) carried out a randomized clinical test that early predictive biomarkers like [TIMP-2]x [IGFBP7] identified patients at risk for AKI and preventive treatments were used to reduce AKI occurrence. Early diagnosis of AKI using urine biomarkers after non-cardiac surgery allows for the initiation of renal protective measures, including CRRT, a new approach for assessing renal function in critically ill patients (Clark et al., 2017). Elevated TIMP-2-IGFBP-7 levels are also predictors of negative results in a variety of clinical situations, including dialysis, death, or development to severe AKI in patients with septic shock (AUC: 0.72); AKI in patients following major surgery (AUC: 0.85); and AKI in platinum-treated patients in the ICU (Casas-Aparicio et al., 2022). These findings show that IGFBP-7 and TIMP-2 have powerful diagnostic properties. The US Food and Drug Administration (FDA) has approved TIMP2-IGFBP7 to predict AKI in critically ill adults within 12 h (Guzzi et al., 2019). However, the presence of proteinuria, urinary albumin >125 mg/dl interferes with the test results, and >3,000 mg/dl negates the test results, as does a urinary bilirubin concentration >7.2 g/dl (Vijayan et al., 2016). Therefore, the approval of the new biomarker combination [TIMP-2]-[IGFBP7] represents a significant step forward in the investigation for a reliable and accurate method of identifying early kidney injury. Early kidney disease interventions will be critical in translating AKI biomarkers advances into major improvements in clinical outcomes. Additional trials are required to study the function of this biomarker in preventative efforts and interventional trials to evaluate its efficacy in enhancing AKI results.

## Other novel biomarkers

### Clusterin

Clusterin is a 75–80 kDa molecular weight heterodimeric glycoprotein. Clusterin is only found in trace amounts in normal kidneys, primarily in the intima of the renal arteries and renal tubules. Clusterin expression increases in acute kidney injury, mostly demonstrating anti-apoptotic effects, and is related with lipid utilization, cell aggregation and adhesion. Clusterin has been found to suppress apoptosis in human renal proximal tubular epithelial HK-2 cells via an extracellular pathway, and to protect renal cells (Wang et al., 2016). Its expression is decreased during glomerular lesions, which exacerbates post-ischemic renal injury and proteinuria. After the ischemia-reperfusion injury, mice lacking Clusterin demonstrate the development of renal inflammation and fibrosis (Guo et al., 2016). Clusterin was expressed in dedifferentiated renal tubular epithelial cells, and Clusterin levels considerably raised in the kidney and urine in an early kidney injury model, according to Vaidya et al.(2008) Gentamicin administration to Lewis mice resulted in increased urinary NAG and Clusterin on day 4, as well as a 10-, 116-, and 3-fold rise in renal tissue, urinary, and serum Clusterin on day 9, respectively (Aulitzky et al., 1992). mRNA expression of renal Clusterin was raised 8.5-fold in mice with renal lesions compared to controls, consistent with increased serum creatinine, and urinary levels correlated with the degree of toxic renal damage (Hidaka et al., 2002). Clusterin and KIM-1 are two of the earliest markers of proximal tubular injury, with levels rising within 1 h of injury, far before serum creatinine. In contrast to CysC and KIM-1, Clusterin predicts drug-induced AKI in adults well (AUC:0.86) (Da et al., 2019). Clusterin was found to be superior to conventional indices for the diagnosis of proximal tubular injury by Dieterle et al., indicating a high diagnostic value not only in severe acute kidney injury. It can also be noticed in early stages, when there is no histopathological evidence yet, showing that it can reflect early and mild lesions (Dieterle et al., 2010). Regarding the localization of urinary Clusterin injury, most research has demonstrated that Clusterin expression is higher in tubular injury but not in glomerular injury, implying that the amount of Clusterin may be quantified to detect whether the injury occurred in the tubules or glomeruli (Hidaka et al., 2002). However, in a recent study, Clusterin outperformed renal function indicators targeting both glomeruli and tubules in the assessment of subclinical AKI, indicating that it appears to be a promising marker of renal injury, covering both tubular and glomerular injury (Musiał et al., 2020), and thus Clusterin remains relevant for the detection of not only tubular but also glomerular injury significance. Clusterin has received little attention in human, and its ability to be a marker of dual renal function and renal injury in a wider patient population has yet to be determined.

### Proenkephalin a 119-159(Penkid)

Proenkephalin A 119–159(Penkid) is a 5 kDa peptide that is identical to the precursors of met-enkephalins and leu-enkephalins and is considered to be an alternative marker of the unstable endogenous opioid peptide enkephalins (Hollinger et al., 2018). Enkephalins participate in a variety of physiological processes through binding to opioid receptors, including the γ-opioid receptor, which is expressed in a number of tissues but is especially dense in renal tissues (Denning et al., 2008). Penkid has a long *in vivo* half-life, is stable after collection, is not easily catabolized, and its levels are unaffected by age or gender. Furthermore, it is not a plasma-bound protein and is only filtered solely in the glomerulus (Lima et al., 2022). Penkid plasma concentrations have been observed to correlate positively and inversely with measured glomerular filtration rate (GFR) (Beunders et al., 2020), and high Penkid values appear to represent a more important risk status than low eGFR values, as Penkid predicts 28-days mortality but eGFR does not, and it is therefore considered to be a biomarker of glomerular filtration injury or a biomarker of renal function. Penkid was found to identify AKI 48 h before serum creatinine, and its specificity remained high in the context of major inflammation-driven sepsis, since Penkid remained at very low concentrations in patients with sepsis who did not have renal failure (Rosenqvist et al., 2019). Penkid levels raised with increasing severity of sepsis in Kim et al. (2017) cohort study of 167 patients, with a cut-off value of 154.5 pmol/L with an AUC of 0.73 for the diagnosis of AKI, and Penkid was found to be significantly higher in patients with severe AKI in Camila Lima et al., 2022 study before liver transplantation. Patients had significantly higher AUC of 0.69 (CI 0.54–0.83), cut-off value of 55.30 pmol/L, sensitivity of 0.86, specificity of 0.52 and accuracy of 0.75, and at 48 h after liver transplantation, the performance of Penkid in determining severe AKI was unchanged with an AUC of 0.83 (CI 0.72–0.94), a cut-off value of 119.05 pmol/L, a sensitivity of 0.81, a specificity of 0.90 and an accuracy of 0.84 (Lima et al., 2022). Moreover, Penkid has shown promising results in monitoring renal function in acute patients, especially those with sepsis. Penkid has added value in monitoring renal function in patients with acute heart failure, in addition to reflecting cardiorenal state following acute myocardial infarction and predicting AKI after cardiac surgery (Hollinger et al., 2018). Penkid and TIMP2-IGFBP7 concentrations were measured at the time of ICU patient admission and showed a correlation with AKI severity, confirming a substantial association of PenKid as a filtration marker with AKI (AUC:0.668). When investigating renal replacement therapy (RRT) as an outcome parameter, in our standard ICU population (n = 60), elevated PenKid levels predicted RRT requirement more accurately (AUC:0.778) than elevated TIMP2-IGFBP7 (AUC:0.678) levels (Gayat et al., 2018). Plasma Penkid concentrations on admission are related to an increased risk of death in AKI and burn patients, and predict 90-days mortality, with Penkid having stronger prognostic value than SCr and SOFA. Patients with subclinical AKI (no diagnosis of AKI but with elevated Penkid) have a higher risk of death than those with low Penkid concentrations (i.e., non-subclinical AKI). Penkid provides clinical relevance in the detection of subclinical AKI (Dépret et al., 2020). Detection of subclinical AKI using PenKid provides better phenotypic analysis of patients who do not meet the current definition of AKI. PenKid’s potential utility is evidence of prognostic impact connected with its level, which would transcend the constraints of the current definition of AKI. Subclinical AKI patients have a greater fatality rate than non-subclinical AKI patients, approaching that of AKI patients. PenKid improves the prognosis of patients who were previously categorized as non-AKI (Dépret et al., 2020). However, there have been fewer studies comparing PenKid to other biomarkers, and it has to be seen whether the combination with other indicators can further improve the characterization and definition of sub-AKI and its association with prognosis.

## Clinical implementation of AKI biomarkers

Although progress has been made in identifying AKI biomarkers, their use in clinical practice has not been widely accepted, and it was recommended at the 23rd ADQI Consensus Conference that injury and functional biomarkers can be combined with clinical information to improve the diagnostic performance of AKI, identify different pathophysiological processes, differentiate the etiology of AKI, and assess AKI severity. The identification of particular kidney injury biomarkers has allowed for a more accurate definition of pathophysiology, site, mechanism, and severity of injury, allowing for a more targeted and individualized treatment plan for each AKI patient. Validated biomarkers can predict the development or progression of AKI and may provide opportunities for intervention (Ostermann et al., 2020). Trials have demonstrated that timely initiation of a preventive strategy, TIMP-2-IGFBP7, after renal injury in patients with positive stress biomarkers is helpful in preventing AKI. The incidence of AKI was lowered by 17% 72 h later. To improve the process of care and patient outcomes, it is also recommended to integrate clinical assessment and validated biomarkers to triage patients and optimize the timing and type of intervention. Negative outcomes are also valuable. For example, critically ill patients with oliguria and urinary TIMP-2-IGFBP7 levels less than 0.3 (ng/mL)2/1000 had no increased risk of developing more severe AKI (Ostermann et al., 2020).

## Conclusion

The need to include markers of injury in AKI risk assessment is becoming more apparent, many biomarkers have been explored for their predictive value in a range of conditions, each with its unique characteristics (Table 1), and several novel biomarkers have been developed, but for the time being, none are totally specific for AKI. Therefore, it is still difficult to introduce these biomarkers into clinical practice, and the use of creatinine as a diagnostic criterion is erroneous; it does not rule out the potential of subclinical AKI. In addition, some specific markers, such as using L-FABP to predict AKI in patients with underlying metabolic syndrome or non-alcoholic hepatic steatosis, and the use of NGAL in patients with sepsis, may be influenced by specific clinical situations. Studies on AKI biomarkers have revealed significant changes in results based on different clinical situations such as cardiac surgery, sepsis, renal transplantation, and contrast nephropathy (Bennett et al., 2008). For instance, in patients undergoing cardiac surgery, NGAL is an initial AKI biomarker and IL-18 is a mid-stage AKI biomarker. In contrast, in nephropathy, IL-18 is not an important AKI biomarker, and the exact mechanisms by which each biomarker changes in different clinical situations have yet to be well revealed, and it has been suggested that different clinical situations should identify specific AKI biomarkers to enhance the initial detection and mediation of subclinical AKI. Different AKI biomarkers can indicate different mechanisms of injury and may be able to differentiate particular aspects of renal function (e.g., tubular injury, decreased filtration rate, etc.). NGAL, a distal tubular injury marker, is elevated when tubular cell structure is injured and serves as an early biomarker of a variety of ischemic, septic, or nephrotoxic kidney ailments. IL-18 is created by proximal tubular cells during kidney damage and has shown potential in diagnosing ATI. KIM-1 is conveyed on the apical membrane of proximal tubular cells following kidney damage and is detectable in urine. Given the added diagnostic dimension that the inclusion of biomarkers may bring, it is reasonable to expect biomarkers to suggest particular therapeutic targets for intervention. As a result, future research should concentrate on mechanisms of injury to broaden understanding of AKI phenotypes based on pathophysiology. Enhanced understanding the underlying biological sequence from renal stress to subsequent subclinical or clinical AKI could lead to therapeutic interventions, drug application or suspension, and improved patient prognosis.

**TABLE 1:** Comparison of characteristics of acute kidney injury biomarkers.

Biomarker	Characteristics/functions	Expression time	Animal research	AUCs of AKI prediction	Sample collections	Limitations
CysC	A cysteine protease inhibitor with a molecular weight of 13 KDa that is freely filtered in the glomerulus and almost completely reabsorbed in the proximal tubule	12–24 h	In the mouse model of sepsis, CysC performed significantly better than SCr and BUN, with a three-fold increase in CysC at 3 h post sepsis compared to baseline (0 h) CysC	0.67	ICU Gaygısız et al. (2016) critically ill infants and children Fang et al. (2018)	Cys-C levels can alter as a result of conditions other than renal filtration (e.g.,use of glucocorticoids, thyroid hormones, and systemic inflammation)
				0.72		
KIM-1	A 38.7 KDa type I transmembrane glycoprotein with immunoglobulin and mucin structural domains	1–12 h	KIM-1 shows upregulation mainly in rodent and human S3 segments, inserts into the apical membrane of the proximal tubule, and persists in the epithelium until recovery	0.83	patients with mild and moderate COVID-19 Yasar et al. (2022)	Vulnerability to diabetes, hypertension and atherosclerotic cerebral ischemia, and inflammatory diseases
NGAL	A 25 KDa protein, a member of the lipocalin superfamily, binds to iron-siderophore complexes, thereby limiting iron uptake by bacteria and exerting a bactericidal effect on the innate immune system	1–12 h	In a transcriptome analysis study in a rodent model, NGAL was found to be one of the fastest-rising genes in the kidney early after tubular injury, especially in the distal tubular segment, and NGAL levels in the urine were significantly elevated within 2 h after ischemia-reperfusion injury in the murine kidney	0.95–0.96	Children with congenital heart disease on NSAIDs after CPB Nehus et al. (2017)	Its threshold value is controversial
				0.96	Pediatric Cardiac Surgery Dent et al. (2007)	
				0.72	Adult Cardiac Surgery Ho et al. (2015) critically ill patients de Geus et al. (2011)	
				0.80	emergency department patients Nickolas et al. (2008)	
				0.95	renal transplant patients Pajek et al. (2014)	
				0.77		
IL-18	an interleukin-1 family pro-inflammatory cytokine generated by monocytes/macrophages and other antigen-presenting cells	1–12 h	IL-18-deficient mice are protected from AKI induced by ischemia-reperfusion injury	0.77	systematic analysis of 11 studies in 2,796 patients Lin et al. (2015)	Lack of suitable cut-off values
				0.8325	CPB Wang et al. (2017)	
				0.82	Pediatric Cardiac Surgery Krawczeski et al. (2011); Zheng et al. (2013)	
				0.59	ICU Nickolas et al. (2012); Nisula et al. (2015) emergency department patients Nickolas et al. (2012); Nisula et al. (2015)	
				0.64		
L-FABP	It is a 14 kDa protein belonging to the fatty acid-binding protein (FABP) family, a potent endogenous antioxidant and one of the key regulators of free fatty acid (FFA) stability in the body	1–12 h	In a cisplatin-induced kidney injury model in mice, elevation of urinary L-FABP levels preceded creatinine elevation over 72 h	0.810	Pediatric Cardiac Surgery Portilla et al. (2008)	Urinary L-FABP may lose its specificity for renal disease in early diabetic nephropathy, non-diabetic chronic kidney disease, polycystic kidney disease and idiopathic focal glomerulosclerosis, when liver disease is also present
				0.720	Adult Cardiac Surgery Lee et al. (2021)	
				0.742	CPB Lee et al. (2021)	
				0.930	ADHF Lee et al. (2021)	
TIMP2·IGFBP7	TIMP-2, a 21 KDa protein with anti-apoptotic and proliferative properties, and IGFBP7, a 29 KDa protein and IGF-1 receptor antagonist, cause tumor suppression and regulate cellular aging by inhibiting kinase signaling.TIMP-2 and IGFBP7 are both inducers of G1 cell cycle arrest	1–12 h	In an experimental study in rats, the combination of TIMP2 and IGFBP7 was more accurate for the diagnosis of AKI than each marker alone	0.682	ICU in COVID-19 Casas-Aparicio et al. (2022)	The presence of proteinuria in the patient, urinary albumin >125 mg/dl will interfere with the test results, and >3000 mg/dl will invalidate the test results, as will the concentration of urinary bilirubin >7.2 g/dl
				0.81	Cardiac Surgery Meersch et al. (2014)	
				0.72	Sepsis Casas-Aparicio et al. (2022) major surgery Casas-Aparicio et al. (2022)	
				0.85		
Clusterin	It is a heterodimeric glycoprotein with a molecular weight of 75–80 kDa, which exhibits anti-apoptotic effects and is associated with lipid utilization, cell aggregation and adhesion	1–12 h	In mice with renal lesions, mRNA expression of renal Clusterin was increased 8.5 times more than in controls, consistent with increased serum creatinine, and urinary levels correlated with the degree of toxic renal damage	0.86	Medicated AKI in adults Da et al. (2019)	There are relatively few human studies, and its ability to be a marker of dual kidney function and kidney injury remains to be determined in studies in larger patient populations
PenKid	A 5 kDa peptide that is identical to the precursors of met-enkephalins and leu-enkephalins and is considered to be an alternative marker of the unstable endogenous opioid peptide enkephalins	1–12 h	—	0.73	Sepsis Kim et al. (2017)	There are fewer studies related to the comparison of PenKid with other biomarkers, while it remains to be explored whether the combination with other markers can further improve the definition of sub-AKI and its association with prognosis
				0.69	Pre-operative liver Transplantation Lima et al. (2022)	
				0.83	Post liver transplantation Lima et al. (2022)	
				0.668	ICU Gayat et al. (2018)	
